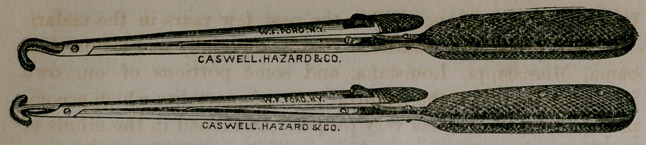# Torsion of Arteries

**Published:** 1874-09

**Authors:** Harry L. Sims

**Affiliations:** New York, Attending Physician to New York State Woman’s Hospital—Outdoor Department


### TORSION OF ARTERIES.

By HARRY L. SIMS, M.D., New York,
*Attending Physician to New York State Woman's Hospital—Outdoor Department.*

Torsion may be said to be an imitation of the means whereby the lower animals, in parturition, by gnawing and twisting the umbilical cord, instinctively arrest its hemorrhage; it is an adaptation of the fact that torn arteries undergo natural hæmostasis more readily and effectually than those that are cut.

The practice of torsion, as a means of controlling hemorrhage from cut arteries, was known to the ancients. It was afterwards pointed out more clearly by Galen, but subsequently passed through a long period of oblivion, and was again revived in the early part of this century, principally through the efforts of French and German surgeons; among whom may be mentioned Amassat, Thierry and Fricke. Each of the operators named had a different method of applying torsion. Amassat practiced what is called *limited* torsion. He used four pairs of forceps: i.e., two ordinary ones, a "forceps a baguettes," and a fixed or torsion forceps. He first seized the end of the artery with the ordinary forceps; with a second pair he isolated the vessel and drew it out half an inch or more from the surface of the wound. The second forceps was then replaced by the torsion forceps, with which the artery was grasped transversely at its extremity. The "forceps a baguettes" was then taken in the other hand, and the vessel was seized transversely by it on a level with the skin. Pressure being now made with the "forceps a baguettes," so as to cut the middle and internal coats of the vessel, the torsion forceps was rotated upon its axis until the end of the artery was twisted off.

Fricke and Thierry practiced free torsion; that is, they simply seized the end of the artery with a forceps, and, drawing it out half an inch or so, twisted it some six or eight times, but did not break off the end.

After these surgeons had practiced torsion for a short time, it again fell into disuse. It was not until about five years ago that it was once more revived by Prof. Syme, as an opposition hæmostatic to the accupressure of Prof. Simpson.

The physiological effect of torsion on the vessels, as observed by Mr. Bryant on the carotids of the horse and on the cadaver are: twisting and division of the external coat, retraction and incurvation of the middle and inner coats with the formation of clot extending from the point of contact of the divided coats to the origin of the first arterial branch above. Another clot may also extend from the same point to the twisted end below. There is no tendency to untwisting in the cellular coat, and its vitality is not impaired in the least degree. The divided and retracted coats are ultimately consolidated by the inflammatory process.

In Guy's Hospital, London, free torsion is the method chosen for the greater part, if not for all, arteries; fixation not being found necessary to prevent the detachment of the artery from its sheath. Whether the artery is fixed or not, the end should not be severed, for it is unnecessary, since it does not untwist when the torsion is done properly and with a proper instrument. It is also unsafe to break off the end, for the clot may be disturbed, and the incurvation of the divided coats destroyed, and thus give rise to hemorrhage.

The advantages of torsion over the ligature are several: 1st. The safety from hemorrhage is greater.

2d. There is a more complete division and a greater retraction of the middle and inner coats.

3d. The twisted cellular coat, and the incurved middle coat, are permanent, as well as temporary, hæmostatics, and for this reason atheromatous arteries are more amenable to control, since in the use of the ligature inflammatory adhesion is the only permanent hæmostatic security.

4th. As the twisted extremity does not slough, there is nothing in the wound to maintain irritation or to favor inflammatory processes, that may bring on hemorrhage.

5th. There is no excuse for failing to secure all the vessels, both large and small, and so the amount of septic material is diminished.

6th. The wound may be more perfectly closed on account of the absence of ligatures, and thus the chances of union by first intention are increased.

The safety and reliability of torsion are well demonstrated by its constant employment in Guy's Hospital, London, where they have not used a ligature for six years. Dr. Tillaux, of Paris, constantly uses torsion, and with great satisfaction and success. I can myself vouch for its efficiency from having seen it constantly employed during my service as Assistant Ambulance Surgeon in the late Franco-Prussian war. We were stationed at Sedan for two months, and, during that fierce and disastrous battle, many amputations were performed, and torsion was substituted for the ligature in a large number of cases. It has been said by some that it would never do to twist so large an artery as the femoral, but in all of our cases we twisted it regardless of its size and formidable appearance. Of the many cases that I saw and dressed daily, I do not remember of but one occurrence of secondary hemorrhage, which was readily controlled by the usual means. I had the care also of some cases in which ligatures had been used, and my torsion cases got well very much sooner than the others.

The first case of torsion of the larger arteries that was done in this country, I believe, was performed by my father, Dr. Marion Sims, about four years ago. Dr. Charles Phelps, of this city, having heard of his large experience at Sedan, and feeling a great interest in the subject, kindly invited him to go to St. Vincent's Hospital, and demonstrate his method of torsion. The first case was amputation of the thigh on a child seven and a half years old. All of the arteries, femoral included, were successfully twisted, and the wound was closed. The child did well, had no unfavorable symptoms, and was shortly after discharged from the hospital.

The other operations following were equally successful, so far as the torsion was concerned. Dr. James R. Wood was present, and was very much pleased with the results, and has since then used torsion to a large extent, twisting both small and large arteries with entire success. Torsion can be applied with great facility and rapidity after a little practice. If the beginner does not at first find that its application is rapid, he should bear in mind that he was once slow in the application of the ligature. Provided the proper instrument be used, I believe that the arteries, as a whole, can be more rapidly secured than with the ligature. The blades of an ordinary forceps are too narrow, and should not be employed; for they cannot bear the strain of the torsion, and they would be liable to tear the vessel. The English forceps are rather clumsy, and are very awkward to handle, as they have a slide catch. My father has modified the English instrument in replacing the slide by a spring catch, and making the blades, which are fenestrated, either curved or T shaped, as shown in the wood-cuts:

The forceps is easily applied over a tenaculum, with which the artery is seized. If the artery is very small, and concealed within muscular tissue, or under a fascia, the surrounding tissues can be twisted with the artery without any difficulty whatever. The number of times that the artery should be twisted varies from four to eight turns of the instrument, this depending upon the size of the vessel. If, in twisting, a slight *click* be heard, which is often the case in the larger arteries, no more twisting is necessary, as that signifies that the middle coat is broken. Care should be taken not to give too many twists, for you may break off the end of the artery.

## Figures and Tables

**Figure f1:**